# Water fluoridation and ethnic inequities in dental caries profiles of New Zealand children aged 5 and 12–13 years: analysis of national cross-sectional registry databases for the decade 2004–2013

**DOI:** 10.1186/s12903-016-0180-5

**Published:** 2016-02-18

**Authors:** Philip J. Schluter, Martin Lee

**Affiliations:** University of Canterbury, School of Health Sciences, Private Bag 4800, Christchurch, 8140 New Zealand; The University of Queensland, School of Nursing, Midwifery and Social Work, Brisbane, QLD 4029 Australia; Canterbury District Health Board, Community Dental Service, Sylvan St., P.O. Box 731, Christchurch, 8024 New Zealand

**Keywords:** Population oral health, Ethnic inequalities, Community water fluoridation, Children, Dental caries, National registry data

## Abstract

**Background:**

Gross and important inequities have historically existed in the oral health profiles of New Zealand children. Following the New Zealand Government’s strategic oral health vision, launched in 2006, nationally collected information from 2004 to 2013 was used to analyze patterns in the prevalence of no obvious decay experience (caries-free) and mean decayed-missing-filled teeth indices over time and by community water fluoridation (CWF) and ethnic classifications in New Zealand children aged 5 years and in school year 8 (generally aged 12–13 years).

**Methods:**

National aggregated data collected from children’s routine child oral health service dental examinations were retrieved, and combined with demographic information from Statistics New Zealand. Children’s CWF status was defined by the public water supply status of their school. Crude and standardized population estimates of caries-free prevalence and mean decayed-missing-filled teeth indices over time were derived. Unweighted linear regression models of main effects and two-factor interactions were investigated by age group.

**Results:**

Dental examination data were available from 417,318 children aged 5 years and 471,333 year 8 children; of whom 93,715 (22.5 %) and 94,001 (19.9 %), respectively, were Māori. Dental examination coverage of Māori children was significantly less than their non-Māori counterparts (approximately 11 % and 14 % for aged 5 and year 8 children, respectively). Regression analysis revealed that caries-free prevalence and mean decayed-missing-filled teeth indices significantly improved over the study period for both age groups. Significant and sustained differences were observed between Māori and non-Māori children, and between CWF and non-CWF exposed groups. However, a convergence of dental profiles between non-Māori children in CWF and non-CWF regions was observed.

**Discussion:**

Significant and important gains in New Zealand children’s oral health profiles appear to have been made over the last decade. Māori children continued to carry a disproportionate oral health burden, even for those in CWF regions. The apparent profile convergence between non-Māori children in CWF and non-CWF regions is noteworthy; although a likely consequence of demographic shifts and unmeasured confounders.

**Conclusions:**

CWF itself did not remove disparities in caries levels between Māori and non-Māori children. Multiple, multi-pronged strategies are needed that overcome the array of factors which disadvantage Māori.

## Background

Globally, and within New Zealand, oral diseases are among the most prevalent of all chronic diseases, and arguably the most preventable [1, 2]. Dental caries is common with an estimated 2.43 billion people (36 % of the world’s population) having caries in their permanent teeth [3], nearly all experience caries at some point in their life [1]; and they are more prevalent in the developed world [4]. Failure to prevent oral diseases has significant personal, societal, and economic costs. In New Zealand alone, expenditure for the treatment of dental diseases is more than NZD$1.1 billion per year [5].

Dental caries severity is measured by counting the number of affected teeth or tooth surfaces; it is cumulative and individuals tend to have a similar rate of increase over time – meaning those with poor oral health early in life are likely to have worse oral health in later years unless effective preventive interventions are introduced [6]. While rates have improved in New Zealand since the 1980s, over 40 % of children still experience dental caries by 5 years of age, and large inequities exist [2]. Māori and Pacific children, children in lower socioeconomic groups, and children residing in non-fluoridated areas have a higher risk of poorer oral health than their peers [2, 7, 8]. Moreover, in the last two decades the national rate of hospital admissions for dental care treatment under general anaesthesia has increased nearly four-fold, from 0.76 per 1000 people in 1990 to 3.01 per 1000 in 2009, with children aged under 8 years having the highest admission rates [9]. Many factors have been identified and implicated with poor oral health status, including nutrition, education, income, ethnicity, insufficient fluoride exposure, oral health behaviours and practices, and irregular dental care [2, 10]. The intricate interplay between these and other individual, social, cultural, economic, and environmental factors can make the development of efficacious oral health preventive strategies challenging.

Community water fluoridation (CWF) has been regarded as being one of the most effective public health intervention for reducing the prevalence and severity of dental caries [11, 12], although a recent Cochrane review challenges this stance [13]. In their review, Iheozor-Ejiofor and colleagues found very strong evidence for the effectiveness of water fluoridation prior to the availability of fluoride toothpaste, however they assert that contemporary evidence of its effectiveness was lacking. Moreover, they found insufficient evidence to determine whether water fluoridation results in a change in disparities in caries levels across socioeconomic status (SES) levels [13]. It has been argued that the exclusion of cross-sectional studies, defined by the Cochrane review methodology, may have led to these findings, and when included, different conclusions might have been drawn [14].

The World Health Organization (WHO) recommends that countries monitor changes in the prevalence of dental caries, and base their recommendations concerning water fluoridation and the use of fluoride toothpastes on these findings [15]. In accordance, since 1990, New Zealand’s Ministry of Health has collected and published data on the oral health of children at 5 years of age and in school year 8 (typically aged 12–13 years) who had received a dental examination during the calendar year. This information is recorded during each child’s routine child oral health service (COHS) dental examination, aggregated, and submitted annually by all New Zealand’s 20 District Health Boards (DHBs) [16]. In New Zealand, approximately 95 % of primary school aged children (aged 5 to 12–13 years) are enrolled in a COHS [17].

The New Zealand Government’s strategic vision for improved oral health, published in 2006, identified the need for oral diseases prevention in early life, with prevention continuing over the life-course [18]. Despite long-term national policy supporting water fluoridation, at the end of 2014 only 54 % of New Zealanders received a fluoridated water supply [19]. A brief current overview and history of CWF and the health effects of water fluoridation in New Zealand can be found in a recent report by the Office of the Prime Minister’s Chief Science Advisor and the Royal Society of New Zealand 2014 [12].

It is recognised that the burden of oral disease and needs of populations are in transition and oral health systems and scientific knowledge are changing rapidly [20]. In order to meet these challenges effectively, public health care administrators and decision-makers need the tools, capacity and information to assess and monitor health needs, choose intervention strategies, design policy options appropriate to their own circumstances, and to improve the performance of the oral health system. In-line with WHO and national priorities, and utilising the most recently available national publically available databases, this study aimed to undertake analyses of the associations between caries prevalence, CWF and ethnicity in 5-year-old and year 8 children for the last decade.

## Methods

### Study design

Secondary analysis of national cross-sectional registry databases combined with Statistics New Zealand population estimates for 2004 to 2013.

### Study population

All children aged 5 years and in school year 8 (generally aged 12–13 years) who had had their oral health status recorded when they received dental treatment in New Zealand’s COHSs between 2004 and 2013. Dental care for children is state-funded in New Zealand from birth until their 18^th^ birthday. Age 5 years and school year 8 corresponds to the first and last year of primary schooling, respectively, and year 8 is the last year when children receive child oral health services. The frequency of children’s dental examinations is based on clinical need and may vary between six and 18 months; this together with workforce and other limitations results in not all children receiving a dental examination within each calendar year.

### Procedure

Each year DHBs submit, to the Ministry of Health, aggregated data on the numbers of children examined, the mean number of decayed, missing, and filled deciduous teeth (dmft) at age 5 years, the mean number of decayed, missing, and filled permanent teeth (DMFT) at year 8, and the number of children without obvious dental caries at age 5 years and at year 8. For the purpose of this paper, we use the terms ‘caries-free’ to define those with no obvious decay experience (that is: no teeth extracted or filled due to caries or carious lesions involving dentine). The dental caries data are collected as part of the provision of routine dental care by dental therapists as opposed to trained and calibrated examiners in an epidemiological study. Extractions and fillings not due to caries and carious lesions not involving dentine are excluded and the diagnosis of caries includes the use of radiography where clinically appropriate. The oral health status is recorded at each child’s first dental examination after their fifth birthday or in school year 8 for 5-year-olds and year 8 children respectively. These aggregated data are stratified by CWF status (with children’s CWF exposure classified by whether their school received a fluoridated water supply or not) and, since 2004, by ethnicity (grouped by Māori, Pacific, and Other). Māori are the indigenous people of New Zealand, with 15 % of the nation’s population identifying with this ethnic group; 7 % of the nation’s population identify as being Pacific, an ethnic group that includes at least 13 distinct languages and cultural groups (predominantly Samoans, Cook Islanders, and Tongans); and the Other group is composed of all other ethnic identifications (predominantly European and Asian) (see: http://www.stats.govt.nz/Census/2013-census/profile-and-summary-reports/infographic-culture-identity.aspx). As people can identify with more than one ethnic group, the reported data used in this study employs the New Zealand standard priority classification [21] – whereby, for people identifying with two or more ethnicities, Māori is placed ahead of Pacific which is ahead of Other (for example a child identifying with both Māori and Pacific ethnicities would be recorded as Māori). Unfortunately, Pacific children’s ethnic identification had been inconsistently collected by two DHBs over the 2004 to 2013 period, thereby reducing the utility of Pacific-specific analyses. In particular, one DHB collected ethnic identification for Māori and Other (including Pacific) only for the period 2004–2006, as did another DHB in 2005. Consequently, Pacific children were combined with the Other ethnic classification, and Māori and non-Māori ethnic subgroups were investigated herein.

The resulting tables of national-level oral health profiles are publicly available in cost-free readily downloadable MS Excel files (at: http://www.health.govt.nz/nz-health-statistics/health-statistics-and-data-sets/oral-health-data-and-stats/age-5-and-year-8-oral-health-data-community-oral-health-service) [16].

Statistics New Zealand provided data on total population estimates of 5 year old children and 12 and 13 year old children by year, and partitions were made of these estimates by Māori and non-Māori groupings. Year 8 population estimates were derived by halving the national total numbers of children aged 12 and 13 years.

### Statistical analysis

Data were imported into Stata version 12.0 (StataCorp, College Station, TX, USA) for all statistical analyses and graphing. Descriptive statistics were calculated and reported for the demographic variables. Statistical investigations of trends over time used unweighted linear regression models. Main effect and two-factor interaction terms were investigated, and statistical significance was assessed based on the Type III score statistic and the Wald’s chi-square test using a manual stepwise elimination approach until the most parsimonious models were identified. An α = 0.05 was used to define statistical significance for all tests. Unless explicitly stated otherwise, absolute rather than relative percentage changes were reported throughout.

For each age group, separate national standardized estimates were made by assuming unexamined children’s dental profiles could be considered exchangeably to those who were examined, matched by age-group, ethnicity, fluoridation status, and year. The number of unexamined children by ethnicity and year was estimated as the differences between the Statistics New Zealand population estimates and the numbers appearing in the COHS database. To estimate the proportion exposed to CWF amongst the unexamined children, and attempt to model changing CWF exposure, for each age group (namely those aged 5 years and in school year 8) and ethnic group (namely Māori and non-Māori) separate second-order linear regression models of the proportion exposed to CWF over time was undertaken for those within the COHS database. The predicted (smoothed) estimates were then applied to the pertinent age and ethnic group of unexamined children to derive estimated numbers of unexamined children exposed and not exposed to CWF for each year. Second-order linear regression models were chosen to capture both linear and quadratic patterns in the data, and to provide smoothed estimates. These standardized analyses attempt to account for any differential examination rates or oral health profile differences between Māori and non-Māori ethnic groups.

### Ethical approval

The study complied with the ethical standards for human experimentation as established by the Helsinki Declaration 1995 (as revised in Edinburgh 2000) and New Zealand’s Health and Disability Ethics Committee (HDEC). HDEC defined this study as minimal risk observational research and it did not require ethics committee review.

## Results

Over the study period, dental examination data were available from 417,318 children aged 5 years and 471,333 year 8 children; of whom 93,715 (22.5 %) and 94,001 (19.9 %), respectively, were identified as being Māori. New Zealand population estimates over this time revealed that there were 587,790 children aged 5 years and 615,485 year 8 children; of whom 149,210 (25.4 %) and 142,645 (23.2 %), respectively, were classified as being Māori.

### Children aged 5 years

The population of 5-year olds and numbers examined from 2004 to 2013 nationally and partitioned by Māori and non-Māori ethnic groupings is presented in Table 1. The total number of children aged 5 years examined increased by over 10,000 during the study period for a population that grew in size by approximately 7000 children. Correspondingly, a significant increase in the proportion of New Zealand children aged 5 years who received dental examinations was observed over this period (*p* = 0.009), with overall coverage increasing by 1.4 % (95 % confidence interval (CI): 0.5 %, 2.4 %) per year. However, examination coverage was not uniform across ethnic groups, with Māori children’s estimated coverage 10.9 % (95 % CI: 7.1 %, 14.7 %) less than non-Māori children. No significant interaction between ethnicity and time was identified (*p* = 0.13), implying that the examination coverage difference between Māori and non-Māori children remained unaltered over the study period.

**Table 1:** Annual estimated numbers of 5-year old children in New Zealand and numbers receiving a dental examination – overall and partitioned by Māori and non-Māori ethnic groupings

	Total			Māori			Non-Māori		
Year	Pop^n^	Exam	(%)	Pop^n^	Exam	(%)	Pop^n^	Exam	(%)
2004	56,790	37,815	(66.6)	14,230	8422	(59.2)	42,560	29,393	(69.1)
2005^a^	58,750	39,173	(66.7)	14,730	8983	(61.0)	44,020	30,190	(68.6)
2006^b^	58,040	39,433	(67.9)	14,670	8711	(59.4)	43,370	30,722	(70.8)
2007^c^	56,240	33,783	(60.1)	13,850	7828	(56.5)	42,390	25,955	(61.2)
2008	56,710	39,240	(69.2)	13,990	8722	(62.3)	42,720	30,518	(71.4)
2009	58,280	43,625	(74.9)	14,440	9967	(69.0)	43,840	33,658	(76.8)
2010	58,080	44,752	(77.1)	14,610	10,069	(68.9)	43,470	34,683	(79.8)
2011	59,290	44,653	(75.3)	15,410	9869	(64.0)	43,880	34,784	(79.3)
2012^d^	61,790	46,668	(75.5)	16,450	10,633	(64.6)	45,340	36,035	(79.5)
2013	63,820	48,176	(75.5)	16,830	10,511	(62.5)	46,990	37,665	(80.2)

^a^West Coast DHB unable to provide dmft data by ethnicity; ^b^West Coast DHB unable to provide dmft data by ethnicity; ^c^Tairawhiti DHB did not supply dmft data and Auckland DHB introduced a new data system which caused major disruption to data collection and reporting; ^d^Hutt Valley DHB and Capital & Coast DHB both excluded a small numbers of children for whom fluoridation status was not reported and Southern DHB did not report data for the 1 January – 20 February period and fluoridation status incomplete for most of the year

**Fig. 1:**
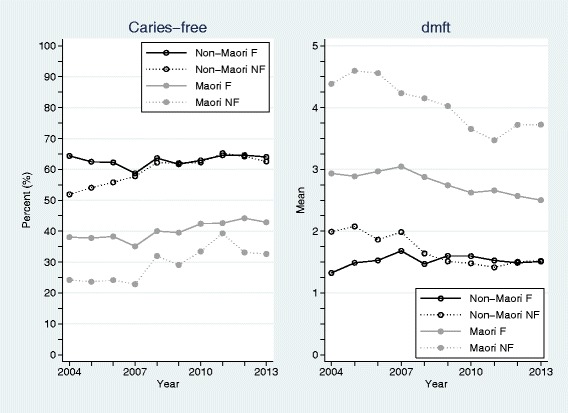
No obvious decay experience (caries-free) percentages and mean dmft for 5-year old children over years 2004 to 2013, partitioned by Māori and non-Māori ethnicities and fluoridated (F) and non-fluoridated (NF) areas

The annual number of 5-year old children in New Zealand who were examined, the numbers caries-free (%), together with their mean dmft appears in Table 2. Figure 1 depicts these measures partitioned by Māori and non-Māori ethnicities and fluoridated and non-fluoridated classifications. In terms of caries-free prevalence, there were significant differences between Māori and non-Māori children (*p* < 0.001), and those children in fluoridated and non-fluoridated areas (*p* < 0.001). There were also significant interactions between ethnicity and fluoridation status (*p* < 0.001), and between fluoridation status and time (*p* = 0.001), but not between ethnicity and time (*p* = 0.17). In 2004, estimated caries-free prevalence for non-Māori children in fluoridated areas was 60.7 % (95 % CI: 58.4 %, 62.9 %), for non-Māori children in non-fluoridated areas was 53.3 % (95 % CI: 51.1 %, 55.6 %), for Māori children in fluoridated areas was 37.8 % (95 % CI: 35.6 %, 40.1 %), and for Māori children in non-fluoridated areas was 23.0 % (95 % CI: 20.7 %, 25.2 %). For children in fluoridated areas, caries-free prevalence improved by an average of 0.5 % (95 % CI: 0.1 %, 0.9 %) per annum over the period of the study; whereas amongst children in non-fluoridated areas, caries-free prevalence improved by an average of 1.4 % (95 % CI: 1.1 %, 1.8 %) per annum.

**Table 2:** Crude and standardized annual numbers (%) of examined 5-year old children in New Zealand who had no obvious decay experience (caries-free) together with mean dmft

	Crude				Standardized^a^			
Year	No. exam	No. caries-free	(%)	Mean dmft	Pop^n^	No. caries-free	(%)	Mean dmft
2004	37,815	19,693	(52.1)	2.11	56,790	29,025	(51.1)	2.18
2005	39,173	20,352	(52.0)	2.24	58,750	30,139	(51.3)	2.28
2006	39,433	20,869	(52.9)	2.15	58,040	30,227	(52.1)	2.22
2007	33,783	17,355	(51.4)	2.27	56,240	28,726	(51.1)	2.27
2008	39,240	22,381	(57.0)	1.98	56,710	31,963	(56.4)	2.03
2009	43,625	24,259	(55.6)	1.97	58,280	32,102	(55.1)	2.01
2010	44,752	25,581	(57.2)	1.90	58,080	32,806	(56.5)	1.94
2011	44,653	26,614	(59.6)	1.82	59,290	34,786	(58.7)	1.88
2012	46,668	27,475	(58.9)	1.84	61,790	35,620	(57.6)	1.93
2013	48,176	27,847	(57.8)	1.86	63,820	36,175	(56.7)	1.93

^a^Up-scaled to national figures based on (i) Statistic New Zealand’s annual population estimates of children by ethnicity, (ii) fluoridation exposure estimates, modelled using a quadratic regression model from the observed annual data over the study period by ethnicity, and (iii) assuming unobserved children’s measurements can be estimated by the observed values for each ethnicity/fluoridation/year combination

Regression analysis of examined 5-year old children’s mean dmft values over the study period also revealed significant differences between Māori and non-Māori ethnic groups (*p* < 0.001), fluoridation and non-fluoridation areas (*p* < 0.001), and significant interactions between ethnicity and fluoridation status (*p* < 0.001), fluoridation status and time (*p* < 0.001), and ethnicity and time (*p* = 0.001). The 2004 estimated mean dmft values for non-Māori children in fluoridated areas was 1.50 (95 % CI: 1.36, 1.64), for non-Māori children in non-fluoridated areas was 2.01 (95 % CI: 1.87, 2.15), for Māori children in fluoridated areas was 3.01 (95 % CI: 2.86, 3.15), and for Māori children in non-fluoridated areas was 4.60 (95 % CI: 4.46, 4.74). Amongst non-Māori in fluoridated areas, mean dmft did not significantly change over the study period (*p* = 0.74). However, mean dmft decreased by: 0.07 (95 % CI: 0.04, 0.09) per annum for non-Māori in non-fluoridated areas; 0.05 (95 % CI: 0.02, 0.08) per annum for Māori in fluoridated areas; and 0.12 (95 % CI: 0.10, 0.15) per annum for Māori in non-fluoridated areas.

Standardized estimates were derived in an attempt to quantify the impact of the differential ethnic examination coverage on national estimates. As Māori children have, on average, lower caries-free prevalence and higher mean dmft, when adjustments are made to correct for their differential coverage pattern, the standardized estimates are uniformly worse than the crude estimates based on the reported data (see Table 2). For caries-free estimates, the standardized values were, on average, 0.8 % less than the crude values (range: 0.3 %, 1.3 %), while for mean dmft, the standardized values were, on average, 0.05 higher than the crude values (range: 0.00, 0.09).

### Year 8 children

The population of year 8 children and numbers examined from 2004 to 2013 nationally and partitioned by Māori and non-Māori ethnic groupings is presented in Table 3. There was a significant decrease in the population numbers of year 8 children over the study period (*p* = 0.001) and also a significant reduction in the number of examinations undertaken (*p* = 0.007); with approximately 379 (95 % CI: 138, 619) fewer examinations undertaken per year. However, there was no change in the proportion of the year 8 children who received examinations over the study period (*p* = 0.23). Again, examination coverage was not uniform across ethnic groups, with Māori children’s estimated coverage 13.9 % (95 % CI: 12.6 %, 15.2 %) less than non-Māori children. No significant interaction between ethnicity and time was identified (*p* = 0.38), implying that the examination coverage difference between Māori and non-Māori children remained unaltered over the study period.

**Table 3:** Annual estimated numbers of year 8 children in New Zealand and numbers receiving a dental examination – overall and partitioned by Māori and non-Māori ethnic groupings

	Total			Māori			Non-Māori		
Year	Pop^n^	Exam	(%)	Pop^n^	Exam	(%)	Pop^n^	Exam	(%)
2004	64,025	49,456	(77.2)	14,465	9506	(65.7)	49,560	39,950	(80.6)
2005	63,050	48,711	(77.3)	14,305	9707	(67.9)	48,745	39,004	(80.0)
2006	62,385	48,738	(78.1)	14,015	9329	(66.6)	48,370	39,409	(81.5)
2007^a^	61,715	46,592	(75.5)	13,950	9264	(66.4)	47,765	37,328	(78.1)
2008	61,355	47,037	(76.7)	14,085	9520	(67.6)	47,270	37,517	(79.4)
2009	60,610	46,220	(76.3)	14,080	9202	(65.4)	46,530	37,018	(79.6)
2010	60,460	46,740	(77.3)	14,195	9493	(66.9)	46,265	37,247	(80.5)
2011	60,210	44,659	(74.2)	14,225	8874	(62.4)	45,985	35,785	(77.8)
2012^b^	60,650	47,121	(77.7)	14,495	9544	(65.8)	46,155	37,577	(81.4)
2013	61,025	46,059	(75.5)	14,830	9562	(64.5)	46,195	36,497	(79.0)

^a^Auckland DHB introduced a new data system which caused major disruption to data collection and reporting; ^b^Hutt Valley DHB and Capital & Coast DHB both excluded a small numbers of children for whom fluoridation status was not reported and Southern DHB did not report data for the 1 January – 20 February period and fluoridation status incomplete for most of the year

**Fig. 2:**
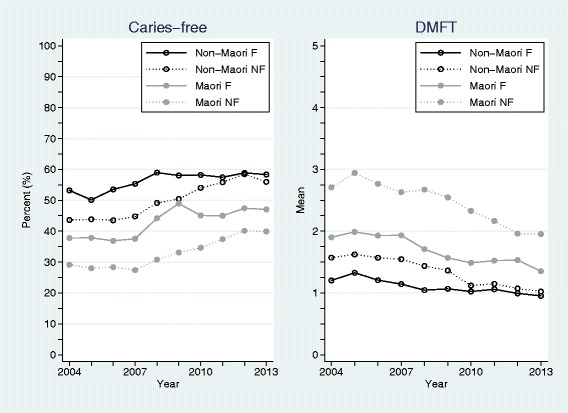
No obvious decay experience (caries-free) percentages and mean DMFT for year 8 children over years 2004 to 2013, partitioned by Māori and non-Māori ethnicities and fluoridated (F) and non-fluoridated (NF) areas

The annual number of year 8 children in New Zealand who were examined, who were caries-free (%), together with their mean DMFT appears in Table 4. As before, Fig. 2 depicts these measures partitioned by Māori and non-Māori ethnicities and fluoridated and non-fluoridated areas. In terms of caries-free prevalence, there were significant differences between Māori and non-Māori children (*p* < 0.001), and those children in fluoridated and non-fluoridated areas (*p* < 0.001). There were also significant interactions between ethnicity and fluoridation status (*p* = 0.01), and between fluoridation status and time (*p* = 0.01), but not between ethnicity and time (*p* = 0.69). The 2004 caries-free prevalence estimates for non-Māori children in fluoridated areas was 51.4 % (95 % CI: 49.4 %, 53.4 %), for non-Māori children in non-fluoridated areas was 42.4 % (95 % CI: 40.4 %, 44.4 %), for Māori children in fluoridated areas was 38.0 % (95 % CI: 35.9 %, 40.0 %), and for Māori children in non-fluoridated areas was 25.3 % (95 % CI: 23.3 %, 27.3 %). For children in fluoridated areas, caries-free prevalence improved by an average of 1.1 % (95 % CI: 0.7 %, 1.4 %) per annum over this period; whereas amongst children in non-fluoridated areas, caries-free prevalence improved by an average of 1.6 % (95 % CI: 1.3 %, 2.0 %) per annum.

**Table 4:** Crude and standardized annual numbers (%) of examined year 8 children in New Zealand who had no obvious decay experience (caries-free) together with mean DMFT

	Crude				Standardized^a^			
Year	No. Exam	No. caries-free	(%)	Mean DMFT	Pop^n^	No. caries-free	(%)	Mean DMFT
2004	49,456	22,573	(45.6)	1.57	64,025	28,801	(45.0)	1.60
2005	48,711	21,569	(44.3)	1.67	63,050	27,637	(43.8)	1.70
2006	48,738	22,276	(45.7)	1.57	62,385	28,216	(45.2)	1.60
2007	46,592	21,737	(46.7)	1.53	61,715	28,692	(46.5)	1.54
2008	47,037	23,997	(51.0)	1.42	61,355	31,105	(50.7)	1.44
2009	46,220	24,079	(52.1)	1.36	60,610	31,255	(51.6)	1.39
2010	46,740	24,890	(53.3)	1.23	60,460	31,771	(52.5)	1.26
2011	44,659	23,993	(53.7)	1.24	60,210	31,972	(53.1)	1.27
2012	47,121	26,370	(56.0)	1.16	60,650	33,445	(55.1)	1.20
2013	46,059	25,060	(54.4)	1.12	61,025	32,923	(53.9)	1.15

^a^Up-scaled to national figures based on (i) Statistic New Zealand’s annual population estimates of children by ethnicity, (ii) fluoridation exposure estimates, modelled using a quadratic regression model from the observed annual data over the study period by ethnicity, and (iii) assuming unobserved children’s measurements can be estimated by the observed values for each ethnicity/fluoridation/year combination

In terms of examined year 8 children’s mean DMFT values, there were significant differences between Māori and non-Māori ethnic groups (*p* < 0.001), fluoridation and non-fluoridation areas (*p* < 0.001), and significant interactions between ethnicity and fluoridation status (*p* < 0.001), fluoridation status and time (*p* < 0.001), and ethnicity and time (*p* = 0.001). The 2004 estimated mean DMFT values for non-Māori children in fluoridated areas was 1.26 (95 % CI: 1.17, 1.36), for non-Māori children in non-fluoridated areas was 1.69 (95 % CI: 1.58, 1.77), for Māori children in fluoridated areas was 2.01 (95 % CI: 1.91, 2.10), and for Māori children in non-fluoridated areas was 2.95 (95 % CI: 2.86, 3.05). Mean DMFT values significantly decreased for all ethnic and fluoridation status combinations over the study period, estimated by: 0.07 (95 % CI: 0.06, 0.09) per annum for non-Māori children in fluoridated areas; 0.07 (95 % CI: 0.04, 0.09) per annum for non-Māori children in non-fluoridated areas; 0.07 (95 % CI: 0.05, 0.09) per annum for Māori children in fluoridated areas; and 0.11 (95 % CI: 0.09, 0.13) per annum for Māori children in non-fluoridated areas.

Making the same assumptions as those used for the 5-year old children, standardized estimates were determined in an attempt to quantify the impact of the differential ethnic examination coverage on national estimates. The standardized estimates were again uniformly worse than the crude estimates based on the reported data (see Table 4). For caries-free estimates, the standardized values were, on average, 0.5 % less than the crude values (range: 0.2 %, 0.9 %), while for mean DMFT, the standardized values were, on average, 0.03 higher than the crude values (range: 0.01, 0.04).

## Discussion

Accumulated evidence from the annually submitted DBH data reveals that population coverage of COHS checks increased over the decade for children aged 5 years and in year 8. Over this time, pre-school enrolments with DHB COHSs have also significantly increased (from 43 % in 2007 to 73 % in 2013) [22]. These increased enrolments and interactions with services are likely to positively influence oral health behaviours and the improved outcomes reported here. However, significant and important ethnic differences in coverage between Māori and non-Māori children remain; differences that failed to significantly diminish over time. The coverage of Māori children aged 5 years and in year 8 were approximately 11 % and 14 % less, respectively, than non-Māori children – and remains unacceptable [2, 23]. The significant reinvestment in COHSs, with its intention of a refocus from treatment to prevention priorities, is squarely aimed at reducing inequalities – which includes increasing access and coverage [18]. While there may be system factors leading to fewer Māori accessing COHSs, evidence shows fewer Māori are recorded in health databases than captured in census data [22], and it is highly likely that misclassification of ethnicity has caused a reduction in the apparent number of Māori having COHS checks. Ongoing efforts in the health sector to improve health ethnicity classification are likely to reduce this discordance in the future.

Patterns in caries-free profiles and changes over the study period were similar for children aged 5 years and in year 8. Overall, caries-free prevalence significantly improved – both in fluoridated and non-fluoridated areas. The average estimated improvements in caries-free prevalence were higher in the non-fluoridated areas compared to fluoridated areas for both age groups. This differential improvement can be explained by lower baseline caries-free prevalence associated with children in non-fluoridated areas, together with the recommendations, health promotion messages, and behavioural changes associated with fluoride toothpaste and other oral health care measures that have occurred over this decade [18, 24]. However, importantly and vexingly, there have been sustained significant differences in caries-free prevalence between Māori and non-Māori children in both age groups over the study period; differences that did not significantly change over time. This is despite the known inequities and risk factors [2, 23], and significant investment to reduce ethnic differentials [18]. With the COHS’s refocus, explicitly aimed at reducing inequalities, the difference between Māori and non-Māori children’s caries-free prevalence it likely to decrease in the future; indeed there is already a suggestion that this is occurring in the more recent national estimates (Figs. 1 and 2).

Another notable feature was the apparent convergence of prevalence estimates amongst non-Māori children in CWF and non-CWF areas. It is likely that a substantial driver of this convergence was due to significant changes within the dynamic and heterogeneous non-Māori groups both within and between DHB regions. In effect, the ecological fallacy – a logical flaw whereby analyses of group data are used to draw conclusions about an individual – may be operating within the non-Māori group. In 2013, when ethnic classification data were available for Māori, Pacific, and non-Māori/non-Pacific (labelled “Other”) groups, Pacific children’s 5-year old caries-free prevalence in non-fluoridated and fluoridated regions was 29.1 % and 37.5 %, respectively, while non-Maori/non-Pacific children’s 5-year old caries-free prevalence in non-fluoridated and fluoridated regions was 63.7 and 70.3 % [16]. As the overwhelming majority of Pacific children were in CWF areas (*n* = 3882, 86.2 %) compared to non-CWF areas (*n* = 623, 13.8 %), this differentially affects the non-Māori fluoridated estimates. Furthermore, Statistics New Zealand’s population projections of 5-year olds reported that Pacific children represented 11.0 % of the age-specific population in 2006, increasing by 0.15 % per year to 13.9 % in 2026 [25]. As Māori children are also having increasing age-specific population proportion representation [25], our non-Māori group is likely to mask important underlying demographic changes. In addition, because there are differences in diagnostic services (principally bitewing radiography) and preventive care between DHBs, and there are significant differences between DHBs in the proportion of children receiving CWF, it cannot be assumed that all children received the same ‘package’ of dental care or that this was evenly distributed between the CWF and non-CWF groups. Further investigation is warranted and individual-level electronic oral health records collected by most DHBs would contain sufficient data for a more detailed analysis.

While caries-free prevalence significantly improved nationally over the study period, it is notable that these rates are below those recently reported in England, Wales and Northern Ireland [26]. This 2013 United Kingdom (UK) survey found that 69 % of 5 year old and 64 % of 12 year old children had no obvious decay experience including visual dentine caries. However, direct comparison is difficult as the New Zealand data are derived from routine dental treatment that includes the use of radiography, whereas the UK survey employed trained/calibrated examiners and different techniques to that of a clinical examination.

When investigating dmft in children aged 5 years and DMFT in year 8 children, an identical statistical pattern emerged to that described in the caries-free exposition – except for the addition of a significant interaction between ethnicity and time in 5-year old children. Here, all groups of 5-year old children had a significant estimated annual decrease in mean dmft over the study period except for the non-Māori children in fluoridated areas whose mean dmft remained largely unchanged. By 2013 it appears that the mean dmft values for non-Māori children were similar for those in fluoridated and non-fluoridated areas; although the values for Māori children were considerably higher and dependent of fluoridated area status. Although non-significant, the gap between mean DMFT estimates for non-Māori year 8 children in fluoridated and non-fluoridated areas also appeared to narrow over time. Again, these results for non-Māori are intriguing and worthy of future investigation, but are likely explained by the ecological fallacy. Future data that reports over consistently defined ethnic classifications (that includes a separate Pacific category) will be necessary to negate this effect. What remains patent is the profound beneficial effects of fluoridation on mean dmft/DMFT levels for Māori.

While this study has a number of salient strengths, including the utilisation of a large, contemporaneous, national databases with excellent coverage and compliance, several important limitations also exist. Fluoridation status is a primary variable of interest; however, children’s exposure to reticulated fluoridated water used a school-based proxy measure which will lead to an unknown level of misclassification. Moreover, the sample is likely to include many new immigrants who had settled in New Zealand within the child’s life-time as well as many New Zealand families who had been mobile, changing regions or suburbs [27]. Thus many children’s exposure to fluoridated water may have been partial or intermittent. These exposure misclassifications are likely to diminish estimated effect size differences between the fluoridation status groups used here. Also, as noted above, the definition of ethnicity itself is problematic [21]. In the 2006 New Zealand Census, 10.4 % of people self-reported more than one ethnic affiliation, with 0.03 % listing six [28]. The difference in definition across registries and agencies hampers investigations of representativeness, and the priority system employed by DHBs is likely to hide vulnerable groups (such as people with both Māori and Pacific ethnic identifications). Moreover, ethnic misclassification is likely to reduce the utility of the reported standardized population estimates. Another important weakness, common to many observational studies using registry data, is the role of unmeasured confounding variables. While age, ethnicity, and fluoridation area were measured and captured, albeit with the caveats described above, omission of other unmeasured determinants of oral health status may introduce potentially important biases into the results reported herein. Unmeasured SES is likely to have the greatest confounding effect, and future analyses demand its measurement and inclusion to yield a better understanding of dental caries profiles with New Zealand. Finally, the completeness of the submitted DHB data was, at times, lacking due to system changes and changes in processes. This is likely to be the primary reason for the relatively large variabilities seen in the various crude estimates (particularly Figs. 1 and 2). For this reason, unweighted regression models were employed and only gross long-term patterns explored. In their current form, it would be disingenuous to use these data to assert meaningful changes between consecutive years or for relatively short periods [29].

## Conclusions

Significant and important gains in children’s oral health profiles appeared to have been made over the last decade, although New Zealand children’s caries experience may still be higher than other countries, such as the UK. However, Māori children, including those in CWF regions, continued to carry an enduring disproportionate oral health burden when compared to non-Māori children. Robson and Reid highlight the structural factors which cause and perpetuate these ethnic differences [30]. Māori do not receive equal or requisite levels of service access, including health. CWF is regarded by many as the most effective public health measure to reduce the burden of dental caries, reducing both its prevalence within a population and its severity in individuals who are affected [11, 12]. It appeared relatively effective in this study, but not a panacea – as Māori children in CWF areas had better profiles than Māori children in non-CWF areas; but still Māori children in CWF areas did worse than their non-Māori counterparts schooled in non-CWF areas. Increasing CWF coverage in New Zealand will only form one part of any solution.

## Abbreviations

Caries-free: no teeth extracted or filled due to caries or carious lesions involving dentine
CI: confidence interval
COHS: child oral health service
CWF: community water fluoridation
DHB: District Health Board
dmft: decayed, missing, and filled deciduous teeth
DMFT: decayed missing, and filled permanent teeth
NZD: New Zealand dollar
SES: socioeconomic status
WHO: World Health Organization
UK: United Kingdom
